# Suppressing the Shuttle Effect of Aqueous Zinc–Iodine Batteries: Progress and Prospects

**DOI:** 10.3390/ma17071646

**Published:** 2024-04-03

**Authors:** Mengyao Li, Juan Wu, Haoyu Li, Yude Wang

**Affiliations:** 1 National Center for International Research on Photoelectric and Energy Materials, School of Materials and Energy, Yunnan University, Kunming 650091, China; 2 Yunnan Key Laboratory of Carbon Neutrality and Green Low-Carbon Technologies, Yunnan University, Kunming 650504, China

**Keywords:** shuttle effect, polyiodide, zinc–iodine battery, iodine

## Abstract

Aqueous zinc–iodine batteries are considered to be one of the most promising devices for future electrical energy storage due to their low cost, high safety, high theoretical specific capacity, and multivalent properties. However, the shuttle effect currently faced by zinc–iodine batteries causes the loss of cathode active material and corrosion of the zinc anodes, limiting the large-scale application of zinc–iodine batteries. In this paper, the electrochemical processes of iodine conversion and the zinc anode, as well as the induced mechanism of the shuttle effect, are introduced from the basic configuration of the aqueous zinc–iodine battery. Then, the inhibition strategy of the shuttle effect is summarized from four aspects: the design of cathode materials, electrolyte regulation, the modification of the separator, and anode protection. Finally, the current status of aqueous zinc–iodine batteries is analyzed and recommendations and perspectives are presented. This review is expected to deepen the understanding of aqueous zinc–iodide batteries and is expected to guide the design of high-performance aqueous zinc–iodide batteries.

## 1. Introduction

With the rapid development of industrialization, the world is currently facing unprecedented environmental pollution and an energy crisis [1,2,3,4,5]. The world’s demand for energy has shown explosive growth, and limited fossil energy sources are no longer able to meet the needs of sustainable development in today’s world [6,7]. The research and development of high-safety, low-cost, high-energy-density, and long-cycle-life energy storage systems has become a major demand for the development of human society [8,9].

In recent years, the rapid development of aqueous batteries based on mild aqueous electrolytes, with the advantages of safety, reliability, environmental friendliness, low cost, and easy processes, is expected to be widely used in consumer electronics and flexible wearable electronics [10,11,12,13]. Zinc metal is an ideal anode for aqueous batteries due to its high theoretical capacity (820 mAh g^−1^, 5854 mAh cm^−3^), low redox potential (−0.76 V vs. SHE), good compatibility and stability in aqueous electrolytes, low price, and wide range of sources [14,15,16]. In the past decade or so of research, cathode materials for aqueous zinc ion batteries have emerged, mainly including vanadium-based compounds [17], manganese-based compounds [18], PBAs [19], organic compounds [20], transition metal group sulfides [21], and other materials. However, the comprehensive performance index of these cathode materials is not high, their capacity is generally lower than 300 mAh g^−1^, and the discharge voltage is generally lower than 0.8 V vs. SHE [22,23]. For these materials, high voltage and high specific capacity also seem to be incompatible. Encouragingly, iodine has become a potential material for aqueous zinc ion batteries due to its many advantages, and it has attracted the attention of a wide range of researchers. First, iodine has the advantage of high natural abundance (50–60 μg/L concentration in seawater) as an cathode [24]. Secondly, the multivalent states of iodine (−1, 0, +1, +3, +5, and +7) give it a great theoretical potential for multi-electron transfer, and therefore iodine has a higher theoretical capacity (two-electron transfer reaction from I^−^ to I^0^ to I^+^: 422 mAh g^−1^) [25]. In addition, iodine can output higher theoretical voltages (I^0^ to I^+^ conversion reaction: 1.83 V vs. Zn^2+^/Zn) [26]. More importantly, unlike the ion embedding and exiting mechanism of traditional “rocking chair batteries”, zinc–iodine batteries rely on a more stable solid–liquid conversion mechanism, i.e., redox of iodine at the cathode and deposition/stripping of Zn at the anode to store capacity, which does not cause serious mechanical and chemical damage to the cathode materials [11,27]. Moreover, the assembly of aqueous zinc–iodine batteries can be completed in air, which is easy to operate and has a broad application prospect. However, due to the inherent disadvantages of iodine monomers (poor electrical conductivity and thermal stability, high activation energy of iodine conversion), the high solubility of polyiodides in aqueous solution, the dendrite growth of zinc anodes, and the occurrence of side reactions resulting in aqueous zinc-iodide batteries still face many problems, such as the shuttle effect of polyiodide, slow redox kinetics and low energy density. which have impeded their further development [28,29]. Among them, the shuttle effect will not only cause the active material in the cathode to gradually decrease, but also the polyiodide will corrode the anode after reaching the anode, thereby increasing the self-discharge effect inside the battery, reducing the cycle life and Coulombic efficiency, resulting in battery performance deterioration [30,31]. Therefore, how to alleviate the shuttle effect has become the focus and hot spot of current research on aqueous zinc–iodine batteries. In the past few years, important progress has been made in the research on inhibiting the shuttle effect. Not only has a series of spatially confined carbon materials been explored, but numerous non-carbon-based materials with adsorption sites or spatially confined structures were also found to have good adsorption effects on iodine analogs, such as MXene, MBene, PBAs, starch, and other organic materials. In addition, a series of new electrolytes, functionalized separators, and modified zinc anodes have been explored, which are expected to accelerate the solution to the shuttle effect problem [24,32,33]. Although researchers have conducted a lot of research on this and achieved many results, aqueous zinc–iodine batteries are still in the initial stage of development and there are few reviews on suppressing the shuttle effect of zinc–iodine batteries. There is an urgent need to summarize and discuss aqueous zinc–iodide batteries in a timely manner. The current status of iodine batteries provides suggestions and directions for the future design and optimization of zinc–iodine batteries. Although researchers have conducted a lot of studies on this and achieved numerous results, the aqueous zinc–iodine battery is still in the primary stage of development, and there is an urgent need to summarize and discuss the current status of the aqueous zinc–iodine battery in a timely manner so as to provide suggestions and directions for guiding the design and optimization of the zinc–iodine battery in the future. More importantly, there are currently few reviews on suppressing the shuttle effect in aqueous zinc–iodine batteries, and most of them describe and summarize from the perspective of specific materials [34,35,36]. In contrast, this review summarizes the current measures to suppress the shuttle effect in aqueous zinc–iodide batteries from a strategic perspective, which can provide readers with a more macroscopic understanding of the material design concepts and measures.

Starting from the basic configuration of zinc–iodine batteries, this article introduces the energy storage mechanism of aqueous zinc–iodide batteries during the charge and discharge process and introduces the triggering mechanism of the shuttle effect faced by current aqueous zinc–iodide batteries. Combined with the latest results, the efforts made by researchers to alleviate the shuttle effect from the cathode, anode, separator, and electrolyte are summarized and analyzed. Finally, some suggestions and directions for the development of aqueous zinc–iodide batteries are provided based on the current research progress and problems, aiming to deepen the readers’ understanding of aqueous zinc–iodide batteries and to promote the development of aqueous zinc–iodide batteries.

## 2. Electrochemistry of Zinc–Iodide Batteries

### 2.1. Zinc–Iodine Battery Configuration

**Figure 1:**
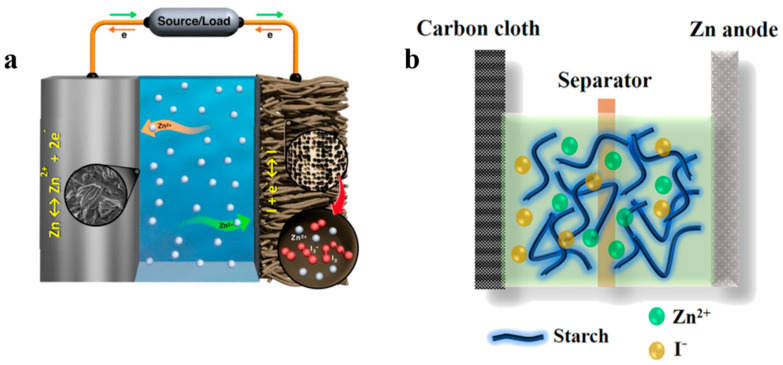
(**a**) Schematic diagram of a zinc–iodide battery with I_2_ as iodine source. Reproduced with permission [37]. Copyright 2017, American Chemical Society. (**b**) Schematic diagram of a zinc–iodide battery with I^−^ as iodine source. Reproduced with permission [41]. Copyright 2017, American Chemical Society. Copyright 2022, John Wiley and Sons.

A typical aqueous zinc–iodide battery is composed of four parts: anode, cathode, electrolyte and separator. Depending on the source of iodine, zinc–iodine batteries can be divided into two categories. In the first category, the iodine is mainly derived from I_2_ anchored in the cathode material [37]. It is worth noting that, in order to prevent the diffusion of I_2,_ current research mainly focuses on injecting I_2_ into the interior of the host material by melt diffusion [38], solution adsorption [39], or electrodeposition [40] and assembling an aqueous zinc–iodine battery with this as the cathode, as shown in Figure 1a. The second category is iodine derived from catholyte or anolyte (Figure 1b) [41], usually in the form of I^−^ or I_3_^−^. For batteries where iodine comes from catholyte, the cathode is usually prepared from a material that can adsorb I^−^ or I_3_^−^, and then the catholyte containing iodine (I^−^ or I_3_^−)^ is dropped or soaked into the cathode. For batteries where iodine (I^−^ or I_3_^−)^ comes from the anolyte, the battery is usually formed by soaking the separator with an iodine-containing salt solution.

### 2.2. Energy Storage Mechanism

The traditional “rocking chair battery” mainly relies on the embedding and de-embedding of ions for energy storage. The de-embedding of ions may cause the volume expansion of the cathode material, which may even cause the structural collapse of the cathode material as the cycling process proceeds, resulting in a poor cycle life of the battery [42]. The energy storage mechanism of the zinc–iodine battery is different, as it relies on a more stable conversion reaction mechanism: the conversion of Zn^2+^/Zn at the anode and the conversion of iodine substances on the surface of the cathode for energy storage [43,44]. The energy storage mechanism of the two types of zinc–iodine batteries is still in its infancy. The energy storage mechanism varies according to the different configurations.

The first type of zinc–iodine battery uses the host-material-anchored iodine monomers as the positive material; in the process of discharge, part of I_2_ convert into I^−^ after obtaining electrons and then continue to coordinate with excess I_2_ to produce I_3_^−^, I_5_^−^, and other polyiodide intermediates, and the generated intermediates will further obtain electrons to be converted into I^−^, and the negative part of the conversion is on the conversion between Zn^2+^ and Zn. During charging, then, an inverse redox reaction occurs where I^−^ ions can be oxidized to I_2_ and Zn^2+^ is reduced and deposited to form Zn until the battery reaches the maximum electrochemical voltage. The specific reaction equations during discharge are given below.

Cathode:

(1) $$I_{2}+2e^{-}↔2I^{-}$$

(2) $$I^{-}+I_{2}↔{I_{3}}^{-}$$

(3) $${I_{3}}^{-}+2e^{-}↔3I^{-}$$

Anode:

(4) $${\mathrm{Zn}}^{2}+2e^{-}↔\mathrm{Zn}$$

Overall:

(5) $$\mathrm{Zn}+I_{2}↔{\mathrm{Zn}}^{2+}2I^{-}$$

(6) $$\mathrm{Zn}+{I_{3}}^{-}↔{\mathrm{Zn}}^{2+}+3I^{-}$$

The second type of zinc–iodine battery contains I^−^ or I_3_^−^ in the main body of the electrolyte. There is no consensus on the energy storage mechanism of this type of zinc–iodine battery. The generally accepted view is that during the charging process, I^−^ will lose electrons and be converted into I_2_ and be adsorbed on the positive electrode. The generated I_2_ will continue to combine with the excess I^−^ in the electrolyte and spontaneously react to form I_3_^−^ and I_5_^−^ polyiodide intermediates; the anode is the conversion between Zn^2+^ and Zn. When a reverse voltage is applied during discharging, I_3_^−^ in the electrolyte undergo a reduction reaction, gaining electrons to form I^−^, and the Zn metal loses electrons to be converted into Zn^2+^. The specific reaction equation during the charging process is as follows.

Cathode:

(7) $$3I^{-}↔{I_{3}}^{-}+2e^{-}$$

Anode:

(8) $${\mathrm{Zn}}^{2+}2e^{-}↔Zn$$

Overall:

(9) $$I^{-}+{\mathrm{Zn}}^{2+}↔\mathrm{Zn}+{I_{3}}^{-}$$

In addition to I_3_^−^, it is also possible to go on to form higher valence polyiodide (I_5_^−^, I_7_^−^, I_9_^−^, etc.) with I_2_ when an excess of I_2_ is present. Unfortunately, these polyiodides often have poor reversibility, which reduces the practical energy density of aqueous zinc–iodide batteries.

## 3. Shuttle Effect

Aqueous zinc–iodine batteries are considered to be candidate energy storage devices with broad application prospects in the field of energy storage. However, from the internal reaction mechanism, zinc–iodine batteries of both iodine sources produce polyiodide intermediates (such as I_3_^−^, I_5_^−^) during the charging and discharging process, and these polyiodides dissolve uncontrollably into the electrolyte and penetrate the separator. They will be repeatedly oxidized, reduced, and shuttle back and forth between the two electrodes with the help of a concentration gradient. This shuttle effect not only causes the active material in the positive electrode to gradually decrease but also causes a self-discharge reaction with the zinc anode after polyiodide ions arrive at the anode, leading to zinc anode corrosion and surface passivation [45]. Specifically, polyiodides accept electrons on the surface of the zinc anode and are reduced to I^−^, while the zinc anode loses electrons and is oxidized into zinc ions. As the reaction proceeds, polyiodide ions are continuously deposited and dissolved on the anode surface, leading to depletion and corrosion of the zinc anode material, which reduces the stability and cycle life of the zinc anode and also leads to an increase in the self-discharge effect and a decrease in the Coulombic efficiency inside the battery [46]. In addition, the shuttle effect of multiple iodide ions will lead to uneven concentration of the electrolyte inside the battery, affecting the charging and discharging efficiency and capacity decay rate of the battery, which seriously limits the industrialization of aqueous zinc–iodide batteries [47,48].

In addition to this, iodine is inherently thermally unstable, making it susceptible to volatilization even at room temperature, and I_2_ are also electrically insulating, which inevitably leads to slow redox kinetics, high polarization, low energy density, and low iodine availability [49]. In order to solve the above problems, it is necessary to search for materials that strongly interact with iodine and polyiodides, thus improving the thermal stability of iodine and suppressing the shuttle effect of iodine [50]. In order to alleviate the shuttle effect, current researchers have carried out a series of modifications and optimizations from the cathode, separator, electrolyte, and anode and conducted in-depth research on the related mechanisms.

## 4. Design of Cathode Material

As the host material of the iodine element, the cathode material has an important influence on the diffusion of iodine substances due to its morphology, structure, and physical and chemical properties. The ideal host material should contain the following characteristics: (1) Effective physical and chemical adsorption to immobilize I_2_/polyiodide [51]; (2) excellent conductivity for faster electron/ion diffusion [52]; and (3) electrocatalytic activity with accelerated I_2_/polyiodide reaction [53]. Current research is mainly focused on the exploration of new host materials and a series of optimizations of host materials. In particular, improving the electrocatalytic activity of cathode materials to suppress the production of polyiodine intermediates has become a major trend in current research. These measures have achieved certain results in mitigating the shuttle effect of polyiodine intermediates and are expected to build aqueous zinc–iodine batteries with long cycle life. The current measures of anode materials in mitigating the shuttle effect can be summarized as spatial confinement, doping of heterogeneous elements, electrostatic interactions, and the introduction of electrocatalysts. Table 1 briefly summarizes the performance of recent zinc–iodine batteries designed based on cathode materials.

### 4.1. Spatial Confinement

Spatial confinement is one of the most common measures to mitigate the shuttle effect in aqueous zinc–iodine batteries [43,72]. It uses the porous structure of the cathode material to encapsulate the iodine in the adsorption material through the melt diffusion method, solution adsorption method, or electrodeposition method so that the polyiodides are affected by the space resistance during the shuttle process and at a certain level alleviate the shuttle effect to a certain extent. 

#### 4.1.1. Carbon-Based Materials

**Figure 2:**
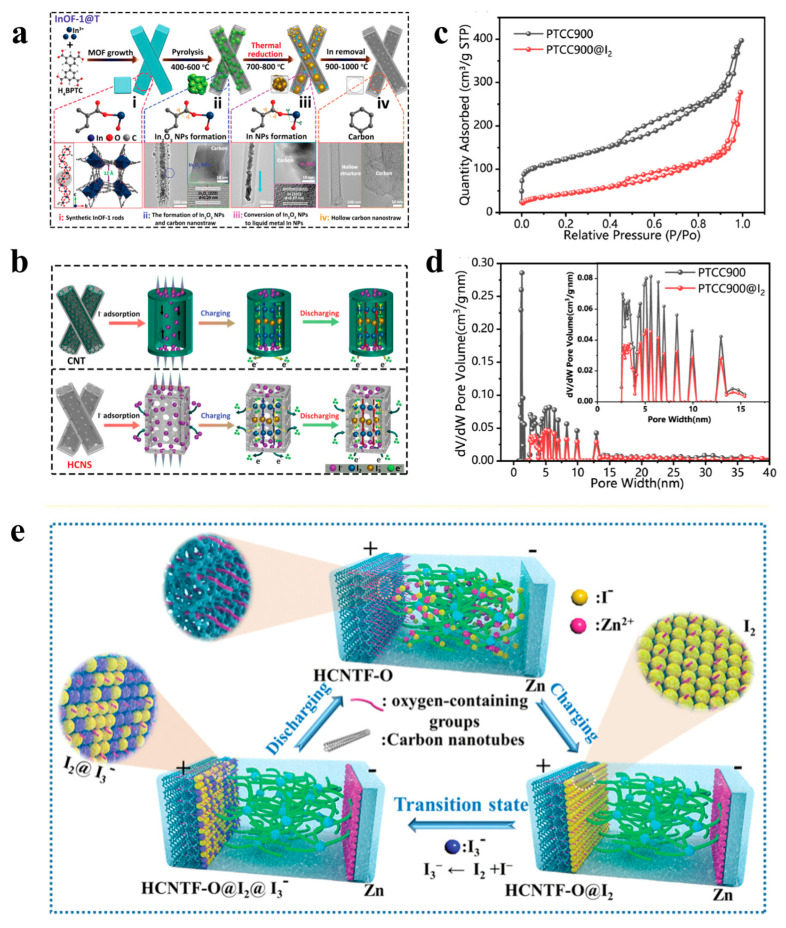
(**a**) Schematic diagram of preparation of HCNS. (**b**) Structural comparison of CNTs and HCNS and schematic diagram of charge storage mechanism. Reproduced with permission [57]. Copyright 2022, Wiley-VCH. (**c**) Nitrogen adsorption–desorption isotherms of PTCC900 and PTCC900@I_2_ and (**d**) pore size distribution curve. Reproduced with permission [82]. Copyright 2023, Elsevier. (**e**) Schematic diagram of ZIDMB operating mechanism. Reproduced with permission [83]. Copyright 2022, Wiley-VCH.

Porous carbon materials (such as carbon cloth, carbon fiber, activated carbon, carbon nanotubes, biomass carbon, MOF-derived carbon materials, etc.) are widely used for iodine adsorption due to the advantages of adjustable structure, large specific surface area, low cost, and the ability to improve the electrical conductivity of iodine [73,74,75]. In the early stages of zinc–iodine battery development, carbon cloth attracted extensive research as a host material for iodine. In 2018, Bai et al. [76] used commercial nanoporous carbon cloth (ACC) as the cathode material and adsorbed the iodine element inside ACC through a simple melt diffusion method to prepare the ACC/I_2_ cathode. Due to the physical constraints of the ACC porous structure and the good affinity of carbon for iodine elements, the thermal stability of I_2_ is significantly improved, resulting in a capacity retention rate of 90% of the battery after 1500 cycles. Carbon nanotubes (CNTs), due to their unique hollow unidirectional tubular structure, can be used as microscopic reaction vessels capable of confining the small molecules of reactive substances for electrochemical reactions. Chai et al. [57] carbonized the indium-based organic skeleton of InOF-1 in an inert nitrogen atmosphere to form indium particles via a redox reaction between indium oxide nanoparticles and a carbon matrix. A porous hollow carbon nanotube (HCNS) was obtained in situ by combining fusion and removal of indium during decarboxylation (Figure 1a). The structural advantages of HCNS over commercial nanotubes (CNTs) are shown in Figure 2b,c. First, HCNS retains a hollow structure similar to CNT so it has more active sites, which can alleviate the volume expansion of the material during charge and discharge. In addition, HCNS also has a porous structure that CNTs do not have, which will greatly shorten the path for ion migration. Therefore, HCNS exhibits better rate performance and cycling stability than typical carbon nanotubes. Compared with traditional carbon precursors, biomass has the advantages of high carbon content, a wide range of sources, rich variety, and low price and is renewable and thus considered an ideal precursor for the preparation of new carbon materials [77,78,79]. Moreover, in the long run, making full use of biomass resources is of great significance to the sustainable development of zinc–iodine batteries. Graded porous carbon materials derived from biomass prepared using ginkgo [55], corn cob [80], and lychee husk [81] as precursors have also been reported for zinc–iodine batteries.

In addition, the size and structure of the pores have an important impact on the spatial confinement effect. By adjusting the pore structure, the confinement effect of the pores can be further enhanced [83]. It has been shown that micropores favor iodine adsorption, while mesopores and macropores favor ion diffusion [84,85]. Therefore, the construction of hierarchical porous carbon with both micropores and mesopores can not only firmly load iodine through the micropores but can also accelerate the reaction through the mesopores, which contributes to the formation of a stable solid–liquid interface and provides an efficient substance transport path for the redox process [51]. Wu et al. [82] used a one-step carbonization method to obtain PTCDA-derived material carbon (PTCC900) as an iodine-loaded cathode material at an annealing temperature of 900 °C without any heteroatom introduction or pore-forming steps. The obtained PTC900 has a typical hierarchical porous structure with a pore size distribution in the range of 1.8 nm–13 nm. Judging from the BET test results, the specific surface area of PTCC900@I_2_ after loading iodine dropped from 422.85 m^2^ g^−1^ before loading to 153.72 m^2^ g^−1^, and pore diameters below 2 nm completely disappeared. This not only demonstrates the successful loading of iodine but also suggests that I_2_ is preferentially loaded in micropores, while mesopores are slightly less able to bind iodine. Thanks to this hierarchical pore distribution within the PTCC900, the shuttle effect is effectively suppressed. As a result, the PTCC900@I_2_ cathode exhibits high capacity (243 mAh g^−1^ at 0.5 A g^−1^), good cycling stability (91 mAh g^−1^ after 50,000 cycles at 5 A g^−1^), and impressive rate capability (242 mAh g^−1^ at 0.1 A g^−1^, 141 mAh g^−1^ at 5 A g^−1^).

To further improve energy storage and extend the life of zinc–iodine batteries, the rational design of a flexible, self-supporting, and binder-free iodine cathode is necessary [64]. Jin et al. [86] used a template method combined with an in situ electrodeposition strategy to construct a flexible aqueous zinc–iodine microbattery (ZIDMB) for the first time. Specifically, the self-supporting films of hydrophilic carbon nanotubes (HCNT-O) containing oxygen functional groups (HCNT-O) and zinc foil were first processed into microelectrodes by laser direct writing, and then they were combined with the prepared cross-linked polyacrylamide/ZnSO_4_/ 1-Methyl-3-n-propylimidazole iodide (CPAM-Zn-I-0.5) polyelectrolyte pairing construct ZIDMB, where the active material of iodine was generated in situ on the cathode during charging, thus significantly simplifying the fabrication and mass production of microdevices. Importantly, due to its carbon nanotube structure and the presence of abundant oxygen-containing functional groups, the introduction of HCNT-O is beneficial to the in situ electroplating of I_2_ and I_3_^−^ on the cathode surface during the charging process, effectively suppressing the shuttle effect of I_3_^−^ and achieving the reversible cycle shown in Figure 2e. It was shown that ZIDMB showed the best cycling performance among all reported aqueous MBs for available aqueous zinc-ion microbatteries (89.2% of the initial value for capacity after the 2600th cycle). In addition, the miniaturized device shows a record volumetric energy density of 1647.3 mWh cm^−3^ and an ultra-high area energy density of 2339.1 μWh cm^−2^. This work provides a new strategy for the future development of high performance portable batteries.

#### 4.1.2. Other Iodine Hosts

**Figure 3:**
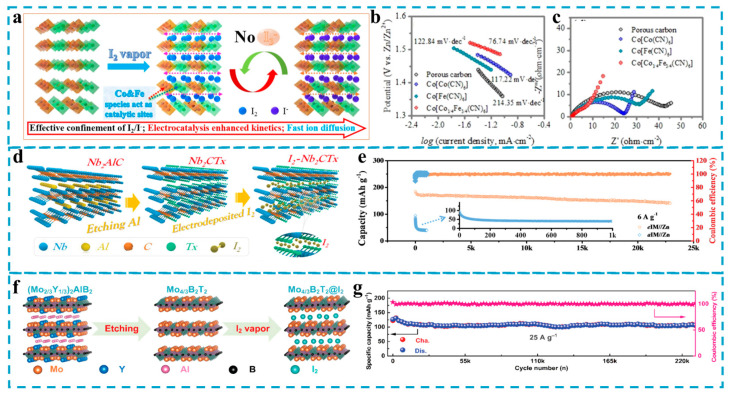
(**a**) Schematic illustration of the preparation of PBAs/I_2_ and the electrochemical process of electrocatalytically active sites with Co and Fe species. (**b**) Tafel diagram of Co[Co_1/4_Fe_3/4_(CN)_6_], Co[Co(CN)_6_], Co[Fe(CN)]_6_ and (**c**) EIS in IRR. Reproduced with permission [58]. Copyright 2020, Wiley-VCH. (**d**) Schematic diagram of the design and fabrication of electrodeposited eIM electrodes. (**e**) Long-term cycling performance of aIM and eIM electrodes at 6 A g^−1^. Reproduced with permission [40]. Copyright 2021, Wiley-VCH. (**f**) Schematic diagram of the synthesis of Mo_4/3_B_2_T_2_@I_2_. (**g**) Long cycle performance at 25 A g^−1^. Reproduced with permission [61]. Copyright 2023, Wiley-VCH.

The success of confining iodine material in microporous and nitrogen-doped porous carbon has also inspired the exploration of other conductive host materials with elaborate spatial confinement structures and adsorption sites, such as Prussian blue analogs (PBAs), MOFs, starch, Mxene, MBene, and other two-dimensional conductive polymeric materials. Among them, Prussian blue analogs can improve the utilization of I_2_ due to their open framework structure and continuous and ordered micropore channels [87,88]. More importantly, they contain electrocatalytic transition metals that can accelerate the redox kinetics of iodine conversion, thereby avoiding the production of polyiodides [59]. There are also reports on PBAs as cathode materials for zinc–iodine batteries. For example, Ma et al. [58] used a three-dimensionally ordered Prussian blue analog Co[Co_1/4_Fe_3/4_(CN)_6_] as the host material for iodine (Figure 3a) and the reactive neutrality of Fe and Co bimetals as active sites in the iodine reduction reaction (IRR) to enhance the reaction kinetics, accelerate the ion diffusion, promote the direct conversion of I_2_ to I^−^, and avoid I_3_^−^ production. Therefore, the Co[Co_1/4_Fe_3/4_(CN)_6_]-I_2_ composite cathode exhibited the lowest potential barrier of 0.47 KJ mol^−1^, the smallest Tafel slope of 76.74 mV dec^−1^ (Figure 3b), and the smallest impedance (Figure 3c), and the assembled zinc–iodine battery provides a high specific capacity of 236.8 mAh g^−1^ at 0.1 A g^−1^. Even at a high current density of 20 A g^−1^, it still has a specific capacity of 151.4 mAh g^−1^, and the maximum power density reaches 22.3 kW kg^−1^, showing excellent performance. 

In the past decades, two-dimensional materials, represented by MXene, have attracted the attention of a wide range of researchers and shown promising applications as conductive carriers [89,90]. Mxene is considered to be an ideal host material for iodine due to its large specific surface area, extraordinary electrical conductivity, abundant surface functional groups, large layer spacing, and high mechanical stability [91,92]. In 2021, Li et al. [40] prepared layered MXene by HF etching and used it as a host material for iodine (Figure 3d). Then, a simple electrodeposition strategy was used to pre-embed the I^−^ in the middle of the MXene nanoscale lamellar channels. The electrodeposited MXene-I_2_ composite electrode (e IM) prepared in this way is highly homogeneous and avoids the problem of inhomogeneous iodine adsorption associated with conventional adsorption methods. More importantly, its nanoscale interlayer spacing significantly limits the migration or leakage of iodine species (I_2_, I^−^, and I_3_^−^), and its shuttle effect is maximally suppressed. The natural affinity of Nb_2_CT_x_ flakes for iodine species (I_2_, I^−^, and I^3^) was also confirmed by DFT theoretical calculations, and the rapid electron supply from Nb_2_CT_x_ flakes accelerated the iodine redox reaction, resulting in a one-step reversible conversion of I_2_ to I^−^. As a result, the MXene-I_2_ composite cathode assembled with the Zn anode to form a battery exhibiting long cycle stability (80% capacity retention after 23,000 cycles) (Figure 3e), high capacity (143 mAh g^−1^), tiny polarization (0.15 V at 18 A g^−1^), and excellent power density (23,505 W kg^−1^).

As a promising post-material for MXene, MBene has also been explored as a host material for iodine-based substances. MBene is derived from the ternary or quaternary MAB phase, where M represents early transition metals, A represents IIIA and IVA group elements, and B represents the element boron [93,94,95]. Zhang et al. [61] first used this transition metal boride (MBene) as a host material for I_2_, which exhibited high rate capability and ultra-long cycling stability when assembled into a battery. Specifically, this work synthesizes layered Mo_4/3_B_2_T_2_ MBene (where “T” represents -O, -OH, and -F terminations) and introduces uniformly in-plane ordered oxygen vacancies within the MBene by a facile approach (Figure 3f). Research shows that the existence of metal vacancies in Mo_4/3_B_2_T_2_ can effectively adjust the charge distribution in the skeleton, which will be beneficial to the adsorption of polar iodine ions and can also be used as a nanoreactor to fix non-polar iodine species. In addition, the Mo_4/3_B_2_T_2_@I_2_ cathode can accelerate reaction kinetics due to its appropriate electronic conductivity. Experimental results and DFT theoretical calculations jointly prove that the Mo_4/3_B_2_T_2_@I_2_ cathode can achieve one-step conversion from I^0^ to I^−^ without producing I_3_^−^, effectively alleviating the shuttle effect. Therefore, the Mo_4/3_B_2_T_2_@I_2_ cathode assembled into a cathode exhibits ultra-long life and high capacity as well as excellent rate capability and tolerance. At a high current density of 25 A g^−1^, after 23,000 cycles, the discharge specific capacity is still as high as 106.8 mAh g^−1^ (Figure 3g); even at an ultra-high current density of 100 A g^−1^, the discharge capacity can reach 80 mAh g^−1^. This work provides a new way to significantly improve the cycle stability and service life of zinc–iodine batteries.

Compared to the weak physical interactions provided by non-polar carbon skeletons, polymers containing polar functional groups such as amino and hydroxyl groups can form stable composite positive electrodes with iodine through bonding and are also considered to be excellent host materials for iodine [96,97]. For example, Qiao’s research team [60] was inspired by the phenomenon that starch turns blue when exposed to iodine and explored the unique interaction between starch action and iodine. Starch is a polymer chain composed of pyranose units, and it has a unique double helix structure formed through intramolecular hydrogen bonds. Therefore, they found that when iodine molecules encounter starch, they enter the double helix chain of starch through bonding and effect, forming a complex of starch and iodine to anchor iodine. And the mechanism of I_5_^−^ dominated I^−^/I_2_ conversion in the presence of starch was demonstrated by in situ Raman. It was confirmed by in situ UV-Vis spectroscopy that I_5_^−^ binds much more strongly to starch than I_3_^−^ in the zinc–iodine battery. Therefore, the presence of starch can significantly inhibit the shuttle effect of polyiodide ions, thereby achieving highly reversible zinc–iodine batteries. After the starch–I_2_ cathode is assembled into a battery, the battery has a Coulombic efficiency of 100% and almost no capacity loss after more than 50,000 cycles, with ultra-long cycle stability. Meanwhile, XPS analysis also showed that the zinc anodic corrosion induced by polyiodide shuttling was significantly suppressed due to the good inhibition of shuttling effect by starch. This research work provides an effective and inexpensive measure for realizing high performance zinc–iodine batteries.

### 4.2. Heteroatom Doping

**Figure 4:**
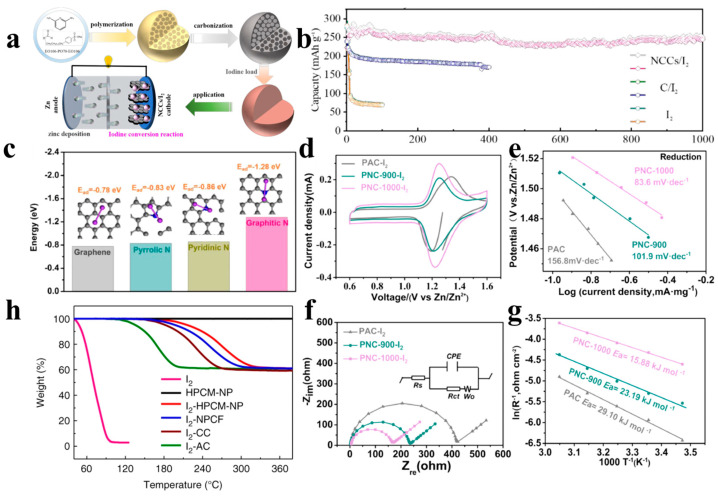
(**a**) Schematic representation of the synthesis of NCCs/I_2_. (**b**) Long cycling performance of NCCs/I_2_ electrodes at 5 A g^−1^. Reproduced with permission [63]. Copyright 2022, American Chemical Society. (**c**) Binding energy between I_2_ and different nitrogen. (**d**) CV curves of zinc–iodine battery with different catalysts at 0.1 mV s^−1^ scan rate. (**e**) Tafel range in the initial discharge stage. (**f**) EIS of zinc–iodine batteries with different electrodes. (**g**) Arrhenius diagrams of the I_2_ reduction process on different electrodes. Reproduced with permission [39]. Copyright 2022, Elsevier. (**h**) Thermogravimetric analysis curves of pure iodine and iodine–carbon composites. Reproduced with permission [104]. Copyright 2017, The Author (s).

Although spatial confinement can mitigate the shuttle effect of the polyiodides to a certain extent, the shuttle effect can still not be suppressed after a long period of cycling through only weak Van der Waals forces (bonding energy of about ~20 meV) between iodine analogs and the host material. Studies have shown that by introducing heteroatoms into host materials (usually carbon materials), they can form XI (X: N, O, P, S, etc.) halogen bonds (bond energy is about 102 meV) with iodine, which can significantly improve the desorption energy of iodine and inhibit the loss of iodine-based active materials [98,99]. In other words, carbon skeletons can regulate the surface charge environment of neighboring atoms through heteroatom doping to form polar surfaces and active sites to anchor polyiodides, which in turn promotes the chemisorption of the polyiodides by carbon skeleton materials [51,100,101,102,103]. For example, Liu et al. [63] prepared N-doped porous carbon nanocages (NCCs) as electrode materials for advanced zinc–iodine batteries through a simple polymerization–carbonization–activation process, as shown in Figure 4a, based on characterization and theoretical calculations. It illustrates that the nanoscale porous structure of N-doped nanocages can provide abundant and powerful polyiodide anchoring sites, inhibit the accumulation of polyiodide in the electrolyte, and promote the reversible transformation between I_2_ and I^−^. Therefore, NCCs/I_2_ composite electrodes demonstrated ultra-high cycling stability with 100% capacity retention after 1000 cycles at 0.1 A g^−1^ (Figure 4b). This is good proof that heteroatom doping is an effective method to improve the performance of zinc–iodine batteries.

In addition, researchers have explored the interaction of different N doping types on iodine. Liu et al. [39] used ZIF-8 as a precursor and calcined it at high temperature to prepare porous carbon (PNC) materials doped with different nitrogens (pyridine nitrogen, graphitic nitrogen, and pyrrole nitrogen). It was found that the type of nitrogen was inextricably linked to the interaction with iodine. Among them, graphite nitrogen has the strongest interaction with iodine, which can inhibit the production of polyiodide intermediates and improve the utilization rate of iodine. This phenomenon can be explained in terms of thermodynamics and kinetics. Firstly, the binding energies of iodine on different carbon bases can be calculated from the thermodynamic point of view to reflect the affinity of different nitrogen sources for iodine, and it can be seen from the calculation results in Figure 4c that the graphitic nitrogen has the smallest binding energy for iodine (−1.28 eV), which suggests that the graphitic nitrogen has stronger interactions with iodine with obvious charge transfer, which will be favorable for the adsorption of iodine by electrode materials. Kinetically (Figure 4d–g), iodine has the smallest activity energy, the smallest Tafel slope, and the largest decomposition energy for polyiodides on graphite nitrogen. These conclusions jointly illustrate that graphite nitrogen can greatly improve the kinetics of the iodine redox reaction. Yu et al. [62] also analyzed the relationship between the type of C-N bond and the magnitude of gravitational attraction of the electrode towards iodine analogues by means of X-ray photoelectron spectroscopy (XPS) and density–functional theory (DFT) calculations and similarly concluded that the graphite-nitrogen functional group (N-Q) has the largest adsorption energy for iodine, while the pyridine–nitrogen functional group (N-6) has the smallest adsorption energy. Importantly, the concentration of heteroatom doping also affects the performance of the battery; the higher the concentration of heteroatom doping, the stronger its chemisorption of iodine will be. Chang et al. [105] wrapped g–C_3_N_4_ in a carbon hexagonal lattice through CSC bond connection and successfully doped a nitrogen–containing g–C_3_N_4_ pyrolyzed species into the carbon matrix in situ. The carbon after high-temperature pyrolysis achieved ultra–high nitrogen content of 13.5%. In addition, the configuration type and doping amount of nitrogen can be freely adjusted through this method. This work used theoretical calculations and experiments to prove that the number of iodine adsorption sites is closely related to the content of pyridine nitrogen, and the number of active sites increased with pyridine N. Moreover, carbon atoms close to the pyridine nitrogen site of adsorbed electrons proved to be the most favorable iodine adsorption sites. Notably, the co-doping of different elements can further enhance the adsorption of I_2_ through charge modulation and defect creation can occur [106]. Therefore, Lu et al. [104] used phytic acid as a phosphorus source doped with polyaniline-modified nonwoven fabric as a precursor and then obtained phosphorus and nitrogen co-doped self-supported layered porous carbon materials (HPCM-NP) by pyrolysis. It can be seen from the thermogravimetric analysis curve shown in Figure 4h that compared with pure iodine, which begins to evaporate at 80 °C, the P and N co-doped porous carbon matrix can significantly improve the performance after adsorbing iodine (HPCM–NP/I_2_). Regarding the thermal stability of iodine, the evaporation temperature of iodine is as high as 200 °C, indicating that iodine has a strong interaction with the carbon skeleton. This strategy of combining porous structure and P and N co–doping not only significantly improves the utilization rate of iodine (high iodine loading mass of 125 wt.%) but also leads to an increase in the number of defects, which can improve iodine adsorption and battery performance. From the DFT theoretical calculation results, it can also be concluded that co-doping of phosphorus and nitrogen will produce synergistic energy and significantly increase the adsorption energy of iodine.

### 4.3. Electrostatic Interactions

**Figure 5:**
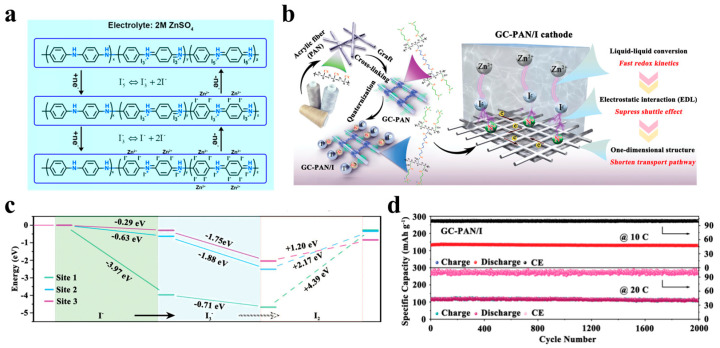
(**a**) Redox mechanism of PANI-I_2_ cathodes. Reproduced with permission [66]. Copyright 2020, American Chemical Society. (**b**) Schematic of the synthesis process of GC-PAN/I and its advantages as a cathode. (**c**) Calculation of the energy of the oxidation process of iodine at active centers 1, 2 and 3. (**d**) Long-term cycling performance of GC-PAN/I cathodes at 10 C and 20 C. Reproduced with permission [47]. Copyright 2022, Wiley-VCH.

Polyiodide can be trapped not only through the spatially confined domains of porous host materials but also with the help of strong electrostatic interactions in the backbone of certain positively charged conductive polymers (e.g., polypyrrole, polythiophene, and polyaniline) [107]. For example, Wu et al. [52] prepared PANI by chemical polymerization using ammonium persulfate as the initiator and aniline as the monomer. Since positively charged nitrogen (–NH^+^=) has a strong electrostatic interaction with anions, it can attract polyiodide (I_3_^−^, I_5_^−^) to balance the charge. Thanks to the “adhesive” effect of PANI on polyiodide ions, the prepared zinc–iodide battery exhibits low overpotential and excellent cycling performance, with a capacity retention rate of 99.9% after 1000 cycles. Similarly, Zeng et al. [66] also used PANI with good conductivity to prepare iodine-complexed PANI–I_2_ cathodes and revealed the redox mechanism of PANI–I_2_ as a cathode (Figure 5a). The redox reaction at the PANI-I_2_ electrode is mainly a transformation between doped I^−^, I_3_^−^, and I_5_^−^. Iodine can form I^−^, I_3_^−^, and I_5_^−^ by reacting with –NH^+^=. These iodide ions act as dopants anchored to the partially oxidized PANI chain and are strongly confined to the main chain of PANI through electrostatic interactions between PANI and iodine substances, thereby effectively inhibiting the dissolution and shuttling of polyiodide in the electrolyte. The successful application of this strategy enabled the battery to achieve a high reversible capacity of 160 mAh g^−1^ and good cycle stability. The capacity retention rate was 79% after 700 cycles at a current density of 1.5 A g^−1^.

In addition to the selection of the specific materials mentioned above as cathode materials for iodine adsorption, quaternization engineering is also an effective means to achieve electrostatic binding of polyiodide ions. Zhang et al. [47] developed quaternization engineering based on the concept of the “electric double layer” (EDL) on a commercial acrylic fiber skeleton. The GC–PAN/I cathode was developed via a facile and processable two–step reaction (Figure 5b) and formed a uniform electrostatic field on a commercially available polyacrylic fiber backbone involving quaternary ammonium ions and polyiodide ions. Studies have shown that this cathode can provide sufficient electrostatic interaction to fix polyiodide on the cathode and reduce the reaction energy barrier, thereby improving the overall performance of zinc–iodine batteries. More importantly, this quaternization engineering is able to create a larger energy barrier between I_3_^−^ and I_2_ (Figure 5c), preventing the production of I_2_, resulting in solution-based iodine chemistry (I^−^/I_3_^−^). As a result, the capacity retention of the zinc–iodine battery with a GC-PAN/I cathode as the p-cathode was as high as 97.24% after 20,000 cycles at 20 C (Figure 5d). In addition, GC–PAN/I cathode demonstrated high-temperature weatherability, reliability, and medium energy density in a variety of complex environments.

### 4.4. Introduction of Electrocatalysts

**Figure 6:**
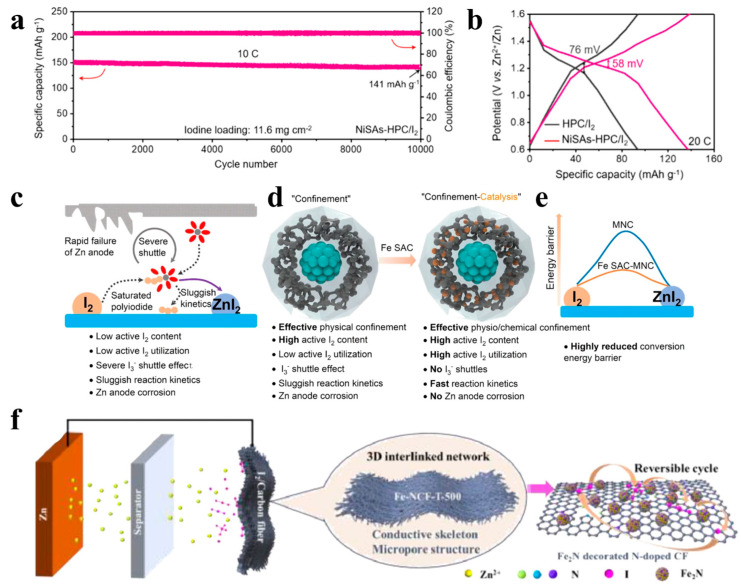
(**a**) Cycling performance of zinc–iodine battery at 10 C. (**b**) Corresponding constant current discharge/charge curves at 10,000th cycle at 20 C. Reproduced with permission [69]. Copyright 2020, American Chemical Society. (**c**) Schematic diagram of iodine redox reaction in aqueous zinc–iodine battery. (**d**) “Restriction + Catalytic” strategy. (**e**) Schematic of the reduction of conversion energy barriers by embedding Fe SACs. Reproduced with permission [38]. Copyright 2023, the authors. (**f**) Schematic diagram of zinc–iodine battery mechanism. Reproduced with permission [70]. Copyright 2023, Elsevier.

The shuttle effect can be effectively mitigated by spatially confined domains and doping with hetero-elements. However, the high activation energy of iodine conversion and the slow redox kinetics due to the insulating nature of iodine cannot be ignored. In order to accelerate the redox kinetics of iodine conversion, the addition of catalytically active metal single-atom catalysts (SACs) such as Ni, Fe, Cu, Co, etc., is the focus of the current research on zinc–iodine batteries. All active metal species in these single-atom catalysts (SACs) exist as isolated single atoms stabilized by the carrier material, which allows for maximum atom utilization compared to bulk metal and nanoparticle catalysts [68,108,109]. Starting from the design of iodine host materials, Ma et al. [69] prepared a hierarchical porous carbon skeleton embedded with Ni single atoms through a template method. The obtained composite material showed excellent cycling stability, with a capacity retention rate of 93.4% after 10,000 cycles at a 10 C (Figure 6a). Impressively, the polarization of the NiSAs-HPC/I_2_ composite after the 10,000th cycle was only 58 mV, much smaller than the 76 mV of the HPC/I_2_ anode (Figure 6b). This is attributed to the fact that hierarchical porous carbon materials can well limit the dissolution of polyiodide intermediates, and at the same time, Ni metal single atoms can catalyze the rapid transformation of intermediate products, thereby jointly suppressing the shuttle effect of the polyiodides. Fe single-atom catalysts have also been shown to be effective electrocatalysts for iodine conversion. Yang et al. [38] proposed a “confinement-catalytic” strategy by embedding Fe single-atom catalysts (SACs) in an ordered mesoporous conducting framework as iodine electrocatalytic host materials. As shown in Figure 6c,d, the ordered porous carbon structure can effectively inhibit the dissolution of I_2_/I_3_^−^/ZnI_2_. At the same time, the Fe single-atom catalytic site significantly reduces the energy barrier of the reaction (Figure 6e) so that the zinc–iodine batteries exhibit fast redox kinetics. Moreover, the zinc–iodine battery exhibits excellent rate capability of 139.6 mAh g^−1^ and ultra-long cycle stability of 50,000 cycles at a high current density of 15 A g^−1^ and a high iodine load of 76.72 wt.%. Although all of the above metal single-atom catalysts exhibit excellent catalytic performance, it is a challenging task to select a suitable SAC to suppress the shuttle effect in zinc–iodine batteries because different metals have different electronic properties that affect the kinetics and thermodynamics of the reaction. In order to find suitable SACs, Yang et al. [68] proposed an I-intoxication mechanism based on DFT theory calculations. They first calculated and compared the catalytic activity and adsorption capacity for I_3_^−^ of eight single-atom catalysts (Cu, Co, Ni, Fe, Mn, V, Zn, and Ti). SACu was found to have the lowest energy barrier in the catalytic reduction of I_2_ to I^−^, which facilitates the one-step conversion of I_2_ to I^−^, and thus not only inhibits the shuttle effect of polyiodide ions but also accelerates the redox kinetics. On the contrary, several SACs are almost inactive due to the strong adsorption of I^−^ to the metal active centers, and the end products of I^−^ will continue to be adsorbed on the metal sites of SAFe, SAV, SAMn, and SATi, thus stifling the catalytic activity of the SACs. In order to further prove its practicability, the authors synthesized Co-doped Ketjen Black (SACo@NKB) and Cu-doped Ketjen Black (SACu@NKB) and compared their properties. The results show that the prepared SACu@NKB is superior to the SACo@NKB cathode in terms of reversible capacity and cycle life. Moreover, SACu@NKB also showed the largest peak reduction current, the smallest Tafel slope, and the smallest activation energy, indicating that SACu has the optimal catalytic activity and the fastest stress dynamics. 

In addition to single-metal catalysts, transition metal oxides, sulfides, nitrides, and carbides are also considered to be excellent electrocatalysts [110,111,112]. Among them, transition metal nitride (Fe_2_N) is used in zinc–iodine batteries due to its high conductivity and excellent catalytic properties. Ding et al. [70] combined the excellent catalytic activity of transition metal nitrides as well as the high specific surface and good flexibility of carbon fibers and synthesized iron-nitride-modified flexible self-supporting porous carbon fibers (Fe-NCF-T-500) as a host material for iodine by electrostatic spinning (Figure 6f). In situ characterization and theoretical calculations show that the modification of highly dispersed iron nitride can effectively adjust the electronic structure of the electrode surface, enhance the charge transfer between iodine and the substrate, and enhance the adsorption of polyiodide by the host material. At the same time, its excellent electrocatalytic activity accelerates the conversion of polyiodine, significantly improves the kinetics of redox reactions, and effectively suppresses the shuttle effect of polyiodine ions, thereby realizing the construction of high-performance zinc–iodine batteries.

In summary, the research on iodine-loaded materials has made exciting research progress. A variety of carbon-based and non-carbon-based materials have been used to efficiently load iodine and suppress the shuttle effect of polyiodide ions. Among them, coupling “adsorption + catalysis, that is, achieving physical confinement and chemical interaction while having good electrocatalytic activity, is the development trend in constructing high-performance aqueous zinc–iodine batteries. Therefore, the following aspects can be considered when designing the cathode material. First, in order to enhance the interaction with the polar iodine, the higher the polarity of the designed cathode material, the better. Secondly, for low-polar carbon materials, the introduction of functional groups containing heteroatoms can transform the weak Van der Waals forces between the subject and the iodine material into strong chemical interactions via halogen bonds. Tirdly, the microstructure of the cathode material is also important. By constructing hierarchical porous carbon with micropores and mesopores, the specific surface area can be increased, while the confinement of iodine and the diffusion rate of ions can be improved. Fourth, explore effective characterization methods to clarify the relationship between the introduction amount of heteroatoms and electrocatalysts and the performance of zinc–iodine batteries. Fifth, in order to improve the redox kinetics of batteries, electrocatalysts can be introduced into the cathode material to lower the reaction barrier and inhibit the production of I_3_^−^. Finally, exploring a simple, low-cost method to prepare high-performance iodine-loaded materials is an important step towards the industrialization of zinc–iodine batteries.

## 5. Electrolyte Optimization

In aqueous zinc–iodine batteries, the electrolyte is an important component [113]. It not only provides a medium for ion transport but can also coordinate with polyiodides through various reaction mechanisms. The selection of electrolytes and the use of additives are crucial to improving the performance of zinc–iodide batteries. They improve the deposition morphology on the electrode surface through interaction with polyiodide ions and provide better conversion kinetics. They work together to suppress the shuttle effect, thereby improving overall battery performance and lifespan. Table 2 briefly summarizes the performance of zinc–iodine batteries based on different electrolytes reported in recent years.

### 5.1. Electrolyte Additives

**Figure 7:**
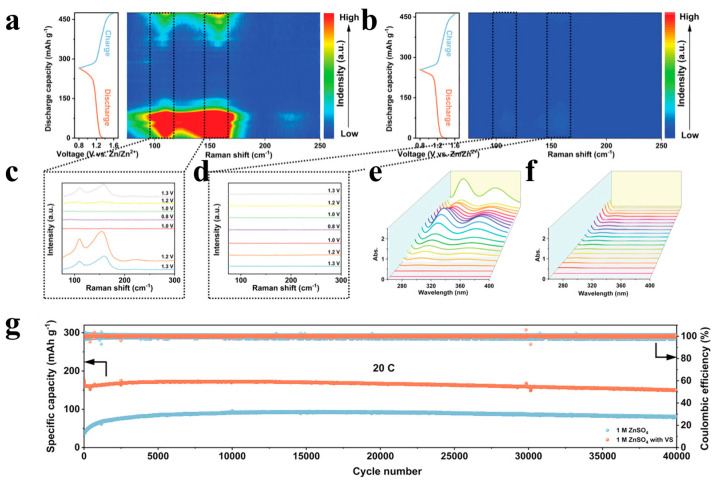
In situ Raman spectra in (**a**,**c**) 1 M ZnSO_4_ and (**b**,**d**) VS electrolytes. In situ UV-visible spectra of (**e**) 1M ZnSO_4_ and (**f**) VS electrolyte during charge and discharge processes. (**g**) Long cycle performance of zinc–iodine battery in 1 M ZnSO_4_ and VS electrolytes. Reproduced with permission [116]. Copyright 2023, Wiley-VCH.

As a simple and effective way, electrolyte additives have achieved significant results in inhibiting the shuttle effect of polyiodines and regulating the deposition behavior of Zn^2+^, but selecting appropriate additives is a challenging task. Zhang et al. [115] found that adding ethylene glycol (EG) to the aqueous electrolyte can produce strong bonds with polyiodides in the solution, thereby inhibiting the shuttle effect of the polyiodides. At the same time, ethylene glycol can also change the coordination environment of zinc, regulate the deposition morphology of zinc, and inhibit the generation of zinc dendrites, thus greatly improving the cycle life of aqueous zinc iodide batteries. In addition, Zhao et al. [116] added vermiculite nanosheets (VS) to a 1 M ZnSO_4_ solution to form a VS suspension electrolyte. Thanks to the interaction of silicon–oxygen bonds on the surface of vermiculite nanosheets with polyiodide, the dissolved polyiodide can be effectively anchored on the surface of vermiculite nanosheets suspended in the electrolyte to suppress the shuttle effect. In order to further study the impact of vermiculite nanosheets on the blocking effect of I_3_^−^ and I_5_^−^ and the electrochemical conversion process, the electrolyte was tested using in situ Raman spectroscopy (Figure 7a–d). In zinc sulfate electrolyte, the peak intensity of I_3_^−^ and I_5_^−^ showed a trend of first increasing and then gradually weakening during the discharge process and gradually became stronger during the charging process, and the peak intensity of I_5_^−^ was significantly higher than that of I_3_^−^. This proves that I_5_^−^ is the main intermediate product and will shuttle to the anode side during the charge and discharge process. For vermiculite electrolyte, no obvious polyiodide Raman signal was detected on the anode side, indicating that the vermiculite suspension electrolyte can well limit the shuttle of polyiodide. The absorbance changes (Figure 7e,f) of the in situ UV test also show a consistent trend with the Raman spectroscopy test. As a result, the cell with VS as electrolyte additive exhibited higher capacity and higher capacity retention than the 1 M ZnSO_4_ electrolyte (Figure 7g).

### 5.2. Eutectic Electrolytes

A eutectic electrolyte is an analog with the electrochemical properties of ionic liquids and with the advantages of good electrical conductivity, excellent electrochemical stability, wide temperature range, simple preparation, and low cost [124,125,126,127,128]. As a new type of electrolyte system, eutectic electrolytes have attracted widespread attention in the field of energy storage [128]. Essentially, the zinc salt eutectic electrolyte is a low eutectic solvent, which is prepared on the principle that a system formed by a low eutectic mixture of Lewis acid–base properties is formed due to a variety of intermolecular interactions that are stronger than its internal reaction when the zinc salt eutectic electrolyte is formed [129,130]. It is worth noting that the eutectic electrolyte is not only an electrolyte with low water activity but also has a completely different solvation structure from the aqueous electrolyte, which is beneficial to inhibiting the shuttle effect of I_3_^−^ [131]. For example, in order to alleviate the problems of low Coulombic efficiency caused by the shuttle effect of polyiodide in aqueous zinc–iodine batteries and uneven deposition and corrosion of zinc anode in aqueous electrolyte, Yang et al. [117] proposed a N-based eutectic electrolyte of methylacetamide. This eutectic electrolyte is prepared by mixing N-methylacetamide, KI, Zn(CF_3_SO_3_)_2_, and water. KI is the only iodine source in the zinc–iodine battery system, and the role of water is to improve the conductivity and viscosity of electrolytes. Unlike the aqueous electrolyte, Zn^2+^ in the eutectic electrolyte has a unique double-shell solvated structure, with the inner layer consisting of I^−^ and Zn(CF_3_SO_3_)_2_ and the outer layer consisting of H_2_O and C_3_H_7_NO. As a result, the electrostatic interactions between the I^−^ in the inner layer and the surrounding ions or molecules will be greatly weakened, making it difficult to combine with the iodine to form I_3_^−^, and thus the generation of I_3_^−^ can be avoided to a certain extent in this manner. DFT theoretical calculations also reveal that the electrolyte is able to increase the Gibbs free energy for the conversion of I_2_ to I_3_^−^, thereby reducing the production of I_3_^−^ and ultimately realizing a direct and complete conversion of I^−^/I_2_ at the cathode interface. To further demonstrate the effective suppression of the shuttle effect by eutectic electrolytes, the researchers used in situ UV-visible absorption spectroscopy to compare the evolution of the valence state of iodine during the cycling process of zinc–iodine batteries based on eutectic electrolytes and aqueous electrolytes. The results show that in the eutectic electrolyte, two slight protrusions appeared at the spectral bands of I_3_^−^ and disappeared during the subsequent cycling process, while in the aqueous electrolyte, the absorbance at the spectral bands of I_3_^−^ increased continuously. Combined with the color changes in cuvettes containing different electrolytes, it can also be found that the cuvette containing eutectic electrolyte remains light yellow, while the aqueous electrolyte appears darker brown. All these phenomena are sufficient to show that the eutectic electrolyte can significantly inhibit the I_3_^−^ produced during the iodine redox process, thus mitigating the shuttle effect.

**Figure 8:**
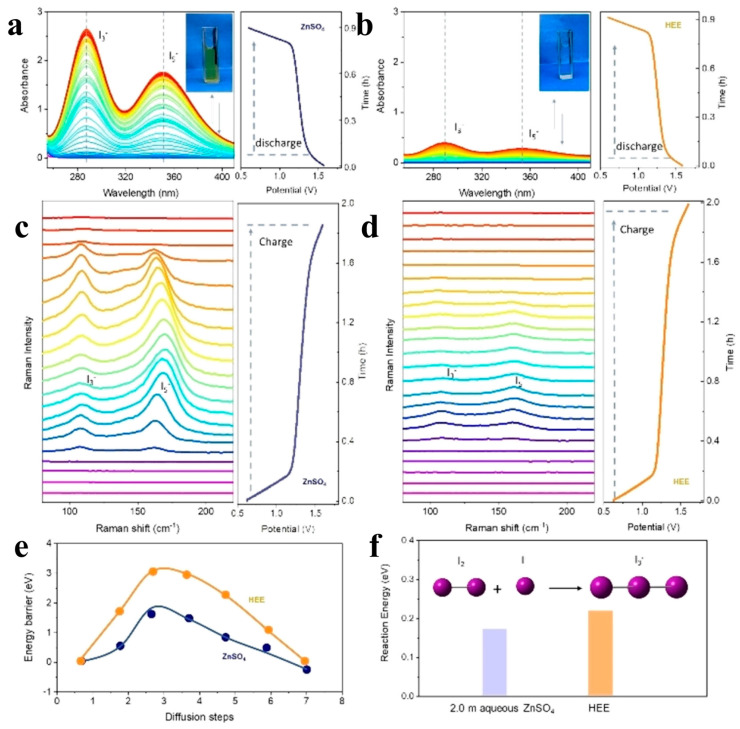
In situ UV-vis spectra of electrolytes in (**a**) ZnSO_4_ and (**b**) HEE aqueous media; inset is an optical image of in situ batteries. In situ Raman spectra of the electrode surface in (**c**) aqueous ZnSO_4_ and (**d**) HEE solutions. (**e**) DFT simulations to compare the diffusion energy of I^−^ in ZnSO_4_ and HEE. (**f**) Energy barriers for I_3_^−^ formation in two electrolytes. Reproduced with permission [118]. Copyright 2023, Wiley-VCH.

In all the current reports, most of the eutectic electrolytes are based on the highly corrosive ZnCl_2_ as well as the more expensive salts such as Zn(CF_3_SO_3_)_2_ and Zn(TFSI)_2_, instead of the milder and inexpensive ZnSO_4_·7H_2_O, which seriously affects the advantages of the aqueous zinc-ion batteries in terms of their low cost and high safety. Therefore, the development of eutectic electrolytes with high safety and low cost is very necessary. Qiao’s team [118] first proposed a series of eutectic electrolytes (HEE) based on ZnSO_4_·7H_2_O by using polyols including ethylene glycol (EG), propylene glycol (PG), and glycerol, which are all low-cost and high-security electrolytes. The evolution of the HEE electrolyte and ZnSO_4_ electrolyte was detected by in situ UV (Figure 8a,b) and in situ Raman spectroscopy (Figure 8c,d), and it was confirmed during the charging and discharging process of the battery that the HEE electrolyte could inhibit the formation of multiple iodide ions, such as I_3_^−^ and I_5_^−^, which slowed down the shuttling effect faced by iodine cathode and effectively improved the performance of zinc–iodine batteries. Moreover, the diffusion energy barrier of I^−^ (Figure 8e) and the formation energy barrier of I_3_^−^ (Figure 8f) in two different electrolytes were calculated by DFT, and the results show that the HEE electrolyte has the largest I^−^ diffusion energy and the smallest I_3_^−^ formation energy, so the HEE electrolyte shows effective confinement of polyiodide. In addition, HEE delays the onset potential of the hydrogen evolution reaction and inhibits the growth of zinc dendrites, allowing the battery to exhibit a Coulombic efficiency (CE) of 99.9% and a cycle life of more than 2000 h, even at −30 °C. It can maintain more than 1000 cycles in harsh environments, showing strong low-temperature resistance. And unlike water/organic mixed electrolytes, HEE is non-flammable due to the high solvation ratio of polyols, maintaining the safety of zinc batteries. 

### 5.3. Quasi-Solid Gel Electrolytes

**Figure 9:**
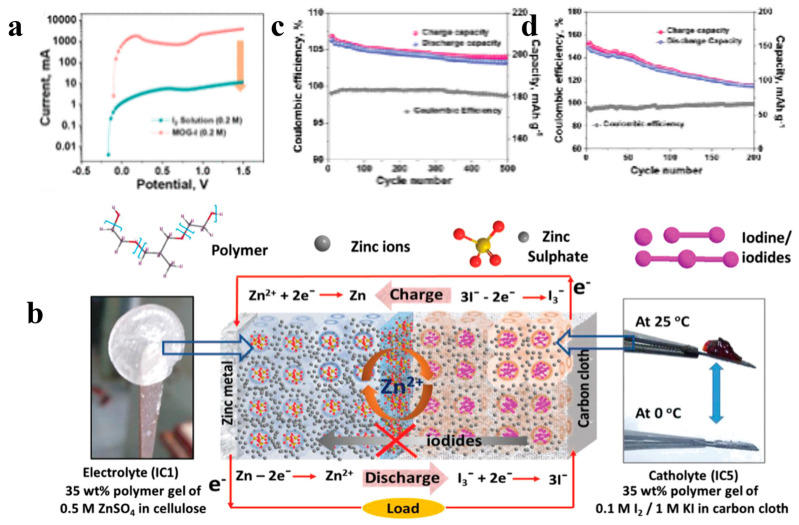
(**a**) LSV of I_2_ solution and MOG-I gel. Reproduced with permission [31]. Copyright 2021, American Chemical Society. (**b**) Schematic diagram of the working mechanism of zinc–iodine battery. (**c**) Long cycle performance of zinc–iodine batteries based on gel electrolytes. (**d**) Long cycle performance of zinc–iodine batteries based on liquid electrolyte. Reproduced with permission [123]. Copyright 2020, Wiley-VCH.

Compared with liquid electrode solutions, quasi-solid gel electrolysis not only can significantly reduce the content of free water, thereby inhibiting the shuttle effect of polyiodide ions and dendrite growth, but also has good flexibility and viscoelasticity, making it suitable for wearable devices in the future [132,133]. For example, Machhi et al. [31] reported an iodine-rich soft metal organogel (MOG-I), which has a sponge-like porous structure and can hold a large amount of iodine-based catholyte. In addition, due to the high viscosity property of MOG, polyiodide ions were firmly adsorbed inside the gel, suppressing the shuttle effect of the polyiodide ions. The diffusion coefficient of iodide in the electrolyte was tested by linear scan voltammetry (LSV), as shown in Figure 9a, to measure the binding ability of the electrolyte to the polyiodides. From the test results, it can be concluded that the diffusion coefficient value of the MOG-I electrolyte is 3.68 × 10^−7^ cm^2^ s^−1^, which is only one-thousandth of the value of the aqueous electrolyte at the same concentration. Thus, the LSV test results similarly demonstrate the inhibition of the shuttle effect by the MOG-I electrolyte.

By designing the gel electrolyte to have ion-selective passability, the limiting effect on the shuttle effect can be further enhanced. Sonigara et al. [123] reported a solid gel reaction with I_3_^−^/I^−^ embedded in a water-based gel in a block copolymer containing highly reactive iodine to limit the diffusion of iodine (Figure 9b). The morphology of this gel changes with temperature and self-assembles into a unique core-shell microcrystalline structure at the critical micelle temperature. Specifically, the core of this microcrystalline structure is composed of hydrophobic PPO blocks in the polymer, and the outer shell is an arrangement of hydrophilic PEOs with a large number of water-rich channels. Moreover, the anions (I^−^/I_3_^−^/SO_4_^2−^) are located at the PPO-rich inner shell or at the core-shell interface, and the cations (Zn^2+^) are located on the water-rich outer shell. Therefore, this unique structure not only limits the long-distance diffusion of large-sized I_3_^−^ but also enables efficient transport of Zn^2+^ in the water-rich channels of the shell. This unique structure of the gel electrolyte also gives the zinc–iodine battery even better performance. In the test of 500 cycles, the zinc–iodine battery using gel electrolyte showed a capacity retention rate of 94.3% (Figure 9c), while the zinc–iodine battery based on aqueous electrolyte experienced rapid capacity fading during the cycle, and after 200 cycles, the capacity retention rate was 49.0% (Figure 9d).

In addition, through the principle of mutual repulsion of the same charge, the design of gel electrolytes with abundant electronegative groups (e.g., hydroxyl, carboxyl, and sulfate groups) is able to mitigate the shuttle effect through electrostatic repulsion. For example, Shang et al. [122] prepared a polyanionic hydrogel electrolyte by ion exchange and zinc-ion-induced cross-linking. This sodium-alginate-based electrolyte not only has the advantages of low price, being green, and environmental protection but also can absorb a large amount of water. This water retention ability allows Zn^2+^ to be transported smoothly inside it while limiting free water from reaching the electrolyte/zinc anode interface, thereby mitigating corrosion on the zinc anode surface. More importantly, this electrolyte contains a large number of negatively charged carboxyl functional groups, which can make it difficult for I_3_^−^ with the same charge to shuttle due to electrostatic repulsion. Due to the many advantages mentioned above, zinc–iodine batteries based on this gel electrolyte exhibit high specific capacity, high Coulombic efficiency, and good cycling stability. 

In short, gel electrolytes show great potential in aqueous zinc–iodine batteries as well as in the wearable sector. However, before preparing the gel electrolyte, the following two issues should be considered. First, a low-cost and environmentally friendly synthetic method is explored to prepare gel electrolytes for industrialization. Second, the prepared gel electrolyte should have high ionic conductivity for fast ion transport and strong interactions with polyiodide ions to suppress the shuttle effect.

## 6. Separator Modification

The separator, an important component of the aqueous zinc–iodine battery, forms a channel for ion migration during the electrochemical reaction [134,135,136]. Conventional separators, such as glass fiber separators and polypropylene separators, have difficulty limiting the shuttling of soluble polyiodides. Therefore, in order to avoid the shuttling effect of polyiodide ions, the separators can be modified and optimized to restrict the polyiodide ions to the positive side. Table 3 briefly summarizes the comparative performance of zinc–iodine batteries based on diaphragm modifications in recent years.

It is worth noting that the modification of the separator does not involve complex electrode design and is a simple and effective method to adjust the interface environment between the anode and the cathode [139]. Generally speaking, highly soluble polyiodide ions are negatively charged in the electrolyte. According to the basic principle of mutual repulsion between similar charges, introducing negatively charged groups in the separator can prevent polyiodide ions from migrating to the zinc anode side. Cation exchange membranes contain fixed charged groups and exchangeable counterions that selectively allow the passage of Zn^2+^ while inhibiting the shuttling of polyiodide ions [141,142]. 

**Figure 10:**
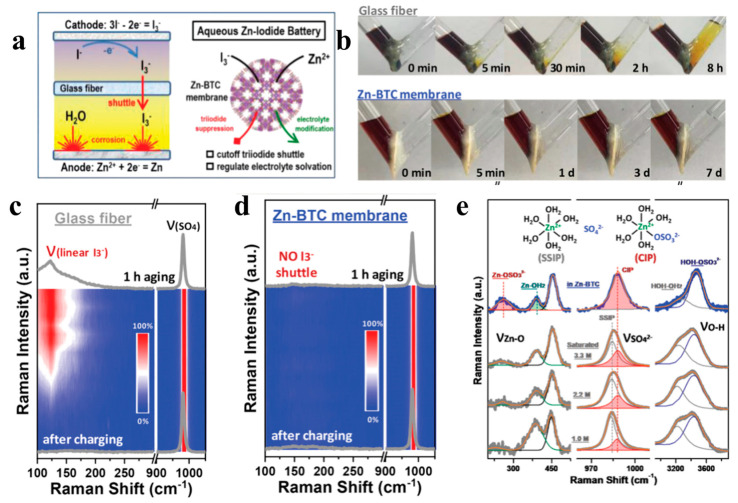
(**a**) Schematic of a zinc–iodine battery with glass fiber (GF) separator and Zn–BTC membrane. (**b**) Optical images of V-glass using GF separator (**top**) and Zn–BTC film (**bottom**). Raman spectra of separators collected from zinc–iodine batteries after aging of (**c**) GF separator and (**d**) Zn–BTC film for 1 h. (**e**) Raman spectra of electrolyte configurations in aqueous ZnSO_4_ solutions (different solution concentrations) and Zn–BTC channels. Reproduced with permission [144]. Copyright 2020, Wiley–VCH.

Due to the expensive price of Nafion membranes (USD 500–700/m^−2^) and low ionic conductivity, developing other ion membrane screens that use pore size to limit the diffusion of I_3_^−^ is also an effective means to suppress the shuttle effect [143]. Metal–organic frameworks (MOFs) are widely used for gas separation and molecular separation due to their rich pore structure. Inspired by this, Zhou’s team [144] proposed a Zn-BTC (Zn_3_(BTC)_2_) membrane (Figure 10a) with high stability and suitable pore size in water as an MOF-based multifunctional ionic membrane screen for zinc–iodine batteries. In order to visually test the barrier effect of different separators on I_3_^−^, Zn-BTC membranes and ordinary GF separators were used in a V-shaped tube, as shown in Figure 10b, and the color of the liquid inside the anode chamber was observed. It can be noticed that the V-tubes with ordinary GF separators start to turn light yellow in the anode liquid chamber after 5 min and dark yellow after 8 h. In contrast, the liquid in the anode chamber remained colorless even after seven days with the Zn-BTC membrane. This intuitively illustrates that the Zn-BTC film can effectively inhibit the shuttling effect of I_3_^−^, thereby reducing the capacity loss of zinc–iodine batteries and the corrosion of zinc anodes. Monitoring I_3_^−^ through Raman spectroscopy can also further find that the I_3_^−^ signal peak in the ordinary GF separator increases with the increase in detection depth, but the Zn-BTC film does not detect the spectrum that increases with the increase in detection depth (Figure 10c,d). Therefore, the test results of Raman spectroscopy also proved that the Zn-BTC membrane can effectively inhibit the migration of I_3_^−^, while the ordinary GF separator cannot. In addition, Raman spectra, as shown in Figure 10e, indicate that the Zn-BTC membrane can also act as a regulating channel to exclude most of the water molecules and regulate the solventized structure of zinc ions, thus inhibiting side reactions such as hydrogen precipitation and corrosion. Unsurprisingly, the zinc–iodine battery based on Zn-BTC membrane shows more excellent Coulombic efficiency as well as longer cycle life than the zinc–iodine battery with ordinary GF separator, with capacity retention still as high as 84.6% after more than 60,000 cycles. In addition, Li et al. [140] reported an inexpensive zeolite molecular sieve (Na_2_(AlO_2_)_12_(SiO_2_)_12_·xH_2_O) which showed a good potential for application when prepared as a separator. Since the pore size of the zeolite porous framework is about 4 Å, it can limit the migration of I_3_^−^ with a size of 5.14 Å in the electrolyte from the cathode to the anode, so the separator can effectively suppress the shuttle effect. As a result, the zinc–iodine battery assembled with zeolite-based diaphragm exhibits excellent Coulombic efficiency as well as ultra-long cycling stability, with 91% capacity retention after 30,000 cycles at 4 A g^−1^. 

Similar to the design of cathode materials, the design of catalytic sites on the separator can also speed up the kinetics of the iodine redox reaction, promote the one-step reaction of I_2_ to I^−^, and avoid the generation of I_3_^−^ [145]. Kang et al. [139] designed a Januas separator based on single-walled carbon nanotubes modified with cation exchange resin and iron metal nanoparticles. The nano-Fe-modified carbon nanotube cathode layer can effectively anchor polyiodide ions and catalyze the redox kinetics of iodide ions, while the cation exchange resin anode layer rich in SO^3−^ groups is beneficial for adsorbing Zn^2+^ and repelling harmful SO_4_^2−^/polyiodide ions, synergistically improving the stability of the cathode and anode interface. As a result, the application of this diaphragm gives the symmetric battery excellent cycling stability, which can be stably cycled for 2500 h and exhibits a high face capacity of 3.6 mAh cm^−2^.

## 7. Anode Protection

Affected by the shuttle effect, the zinc anode will react with polyiodide (I_3_^−^ + Zn→3I^−^ + Zn^2+)^, resulting in corrosion and passivation on the surface of the zinc anode, which ultimately leads to the formation of the passivation layer and the wanton growth of the dendrites, which seriously affects the stability of the zinc anode and the cycle life of the battery [141]. Therefore, appropriate measures should be taken to inhibit the corrosion of zinc anodes by polyiodides in order to construct long-life aqueous zinc–iodide cells. Table 4 briefly lists the electrochemical properties of modified zinc anodes in recent years. It is worth noting that compared to cathode materials, relatively little research has been conducted on zinc anodes, which still needs to be further explored by scholars.

**Figure 11:**
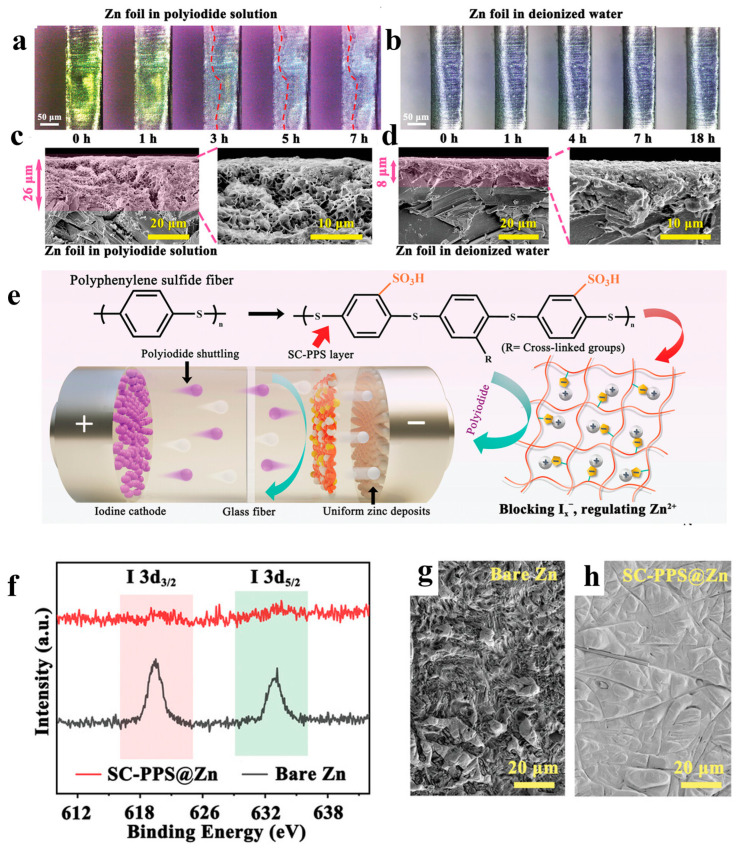
Corrosion process of zinc foil in (**a**) polyiodide solution and (**b**) aqueous solution observed by in situ optical microscopy. Cross-sectional image of zinc foil after 3 days of immersion in (**c**) polyiodide solution and (**d**) deionized water. (**e**) Schematic representation of the synthesis process of SC-PPS and its role in zinc batteries. (**f**) XPS spectra of Zn-I comparing cells with SC-PPS@Zn (SC-PPS layer torn off and bare Zn after 21,000 cycles). (**g**) SEM images of bare Zn plate anode and (**h**) SC-PPS@Zn surface after 1000 cycles. Reproduced with permission [33]. Copyright 2023, Wiley-VCH.

In order to inhibit the side reactions such as corrosion starting from the interface between the electrolyte and zinc anode, Zhang et al. [33] firstly exposed the corrosion mechanism of polyiodide on the zinc anode by exploring the corrosion process of zinc foil in polyiodide solution and aqueous solution. Observation of the surface of the zinc anode by in situ optical microscopy and SEM, as shown in Figure 11a–d, reveals that the zinc anode reacts with polyiodide and forms a raised and honeycombed corrosion layer in the polyiodide solution. In contrast, the surface of the zinc foil in deionized water showed no significant change. This observation confirms that polyiodides accelerate the corrosion and passivation process of zinc anodes, which ultimately leads to dendrite growth and cell failure. Therefore, in order to provide an effective protective layer on the zinc anode surface, the team constructed a sulfonate-rich 1,4 bis(chloromethyl)-2-nitrobenzene cross-linked polyphenylene sulfide (SC-PPS) ion-exchange layer on the surface of the zinc anodes, which mitigates the corrosive effect of polyiodides on the zinc anodes. Specifically, sulfonate can effectively prevent the corrosion of the zinc anode surface by polyiodide ions due to strong electrostatic repulsion with polyiodide negative ions due to the same charge. In addition to this, sulfonate has a good affinity for zinc ions and can form a uniform zinc-rich layer on the surface of zinc in order to promote the uniform deposition of zinc ions and thus inhibit the growth of dendrites. Further, XPS (Figure 11f) and SEM (Figure 11e,h) characterization of the zinc anode surface after 1000 cycles also concluded that the SC-PPS coating could subject the zinc anode surface to polyiodide corrosion and keep it dense and dendrite-free. As a result, the capacity retention of the zinc–iodine battery with SC-PPS coating was 90.2% after 6000 cycles at a current density of 3200 mA g^−1^. This work demonstrates the potential of zinc anode coating for low-cost, high-performance zinc–iodine batteries. 

In addition to constructing coatings on the surface of zinc anodes, preparing artificial SEI films on the surface of zinc anodes by consuming a suitable electrolyte is also a feasible measure to resist polyiodide corrosion. For example, Zhang’s team [147] utilized the coordination chemistry of phytic acid (PA) containing six phosphate groups with zinc ions to prepare an artificial solid electrolyte interface in situ on the surface of a zinc anode for the inhibition of side reactions such as corrosion and dendrites. Based on the dynamic coordination process between phytic acid and zinc ions at the PA-Zn interfacial layer, Zn^2+^ was transferred in an energy-favorable jumping mechanism, and uniform deposition/exfoliation of Zn^2+^ was achieved. In addition, this interfacial coating has a porous channel structure that can effectively regulate the solvation structure of Zn^2+^ dynamically coordinated along the PA skeleton, thus effectively inhibiting the zinc corrosion problem induced by multi-iodide shuttling. Therefore, the obtained zinc anode exhibits good cycle stability, with a capacity retention rate as high as 93.6% after 5000 cycles at 5 C.

## 8. Summary and Outlook

In the past few years, aqueous zinc–iodine batteries have attracted the attention of a wide range of researchers and achieved rapid development due to many advantages such as high safety, low cost, and high theoretical capacity. However, the shuttle effect of polyiodide ions seriously affects their self-discharge performance, Coulombic efficiency and cycle life, which in turn hinders the industrialization of aqueous zinc–iodine batteries. In order to alleviate the shuttle effect, researchers have made great efforts and achieved important results in the design of cathode materials, anode protection, electrolyte regulation, and separator modification. We summarized the innovations of different strategies and the impact on iodine batteries. First of all, due to the shortcomings of iodine, such as poor thermal stability and poor electrical conductivity, researchers have not only developed a series of porous carbon materials with high electrical conductivity, low cost, and large specific surface area as host materials for iodine but have also explored other materials with structurally restricted domains and adsorption sites as iodine monomer host materials, such as MXene, MBene, PBAs, and starch. These materials can anchor iodine through physical confinement effects and increase the steric hindrance for the diffusion of polyiodide ions such as I_3_^−^ and I_5_^−^, alleviating the shuttle effect to a certain extent. However, this spatial confinement is only based on weak Van der Waals forces and cannot stably bond polyiodide ions. Therefore, the researchers further enhanced the interaction between I_3_^−^ and I_5_^−^ with the host material by introducing heteroatoms and electrostatic interactions and significantly improved the desorption energy of iodine. The redox kinetics of iodine conversion was also accelerated by the introduction of an electrocatalyst, which realized a one-step conversion of I_2_ to I^−^ and avoided the production of polyiodide ions. Second, the researchers also carried out a series of optimizations of the electrolyte. The shuttle effect of polyiodide ions in the electrolyte was suppressed by the design of electrolyte additives, eutectic electrolytes, and quasi-solid gel electrolytes. In addition, the separator provides a channel for ion migration, and by modifying the separator, it is possible to confine the polyiodide ions to the cathode side, preventing them from crossing the separator to the anode. Nafion membranes with cation exchange function and ionic membrane sieves with pore size limitation can effectively inhibit the shuttle effect. Not only that, in order to further inhibit the corrosive effect of polyiodides on zinc anodes, the researchers realized the modulation of the zinc anode/electrolyte interface by constructing surface coatings and artificial SEI films. However, the current performance of aqueous zinc–iodine batteries means it is still difficult to meet the demand for practical application, and more efforts are still needed in order to realize the industrialization, commercialization, and application of aqueous rechargeable zinc–iodine batteries. Next, some specific suggestions and directions will be provided for the future development of aqueous zinc–iodine batteries.

1. Cathodes. To construct high-performance aqueous zinc–iodine batteries, “adsorption + catalysis” is the development direction of cathode materials in the future. Judging from the current research results, there is room for improvement in both adsorption and catalysis effects. Firstly, in order to achieve better adsorption effect, functional groups or polymers with polar components can be introduced into materials with spatially restricted domains to stabilize the bonded polyiodide ion intermediates, thus realizing the dual effect of “physical adsorption + chemical adsorption”. It is worth noting that the pore structure, morphology, and surface properties of the adsorbent materials themselves also affect the adsorption effect, but there is a lack of in-depth studies, so the constitutive relationship between them can be further explored in future studies. Secondly, in order to achieve high catalytic activity in cathode materials, the introduction of electrocatalysts is an effective method. However, the current aqueous zinc–iodine cell has only explored the catalytic effect of monoatomic catalysts, Prussian blue analogs containing transition metals, and transition metal family compounds on polyiodide ions while ignoring the potential synergistic effect of bimetallic catalysts, and thus the catalytic effect of bimetallic catalysts on polyiodide ions can be explored in future studies with an understanding of the catalytic mechanism. Finally, to meet the needs of practicalization, a simple and low-cost method to prepare high-performance cathode materials should be actively explored. Moreover, in order to meet the requirements of the wearable field, a cathode material with both high loading capacity and good mechanical properties can be designed.
2. Separators. Due to their unique structure and relatively low cost, MOF materials have shown unique advantages in serving as an ion membrane screen to alleviate the shuttle effect of polyiodide ions. However, the current design of ion membrane sieves is relatively simple. In order to further strengthen the binding of polyiodide ions, more in-depth research on MOF materials can be conducted. First, we can consider introducing organic ligands containing polar functional groups when synthesizing MOF materials. These introduced functional groups can enhance the adsorption of polyiodide ions. Secondly, we can develop new conductive MOF materials to avoid the shortcomings of poor conductivity and poor stability that most current MOF materials still have. In addition, we can develop MOF composite materials to give full play to the advantages of a single MOF material, thereby simultaneously solving multiple problems existing in zinc–iodine batteries. In order to accelerate the kinetics of the iodine redox reaction, combining the chemical adsorption effect with the catalytic effect on the separator is also an effective means to inhibit the shuttle effect of polyiodide ions and accelerate the iodine redox kinetics. It is worth noting that the design of the separator must also consider mechanical properties and weight. The modified separator must not only be light and thin but also have good mechanical properties so that a long-life water system can be constructed without losing energy density in zinc–iodine batteries.
3. Electrolytes. The electrolyte usually has two strategies in inhibiting the shuttle effect, one is to inhibit the production of polyiodide ion intermediates such as I_3_^−^ and I_5_^−^, and the other is to prevent the diffusion of polyiodide ions. For the first way, electrolyte additives can be used. For the second method, the content of free water in eutectic electrolytes and quasi-solid gel electrolytes is greatly reduced, which can reduce the solubility of polyiodide ion intermediates in them so the shuttle effect can be suppressed to a certain extent. However, eutectic electrolytes and quasi-solid gel electrolytes hinder their industrialization due to their low ionic conductivity and large interfacial resistance. Therefore, exploring a polyiodide-free electrolyte with excellent performance and high ionic conductivity is the development direction to inhibit polyiodide shuttle in aqueous zinc–iodine batteries.
4. Anodes. Unlike other aqueous zinc ion batteries, the modified zinc anode of zinc–iodide batteries should not only be able to inhibit the side reactions such as dendrite growth, hydrogen precipitation, and passivation but also give special consideration to the corrosion of the zinc anode due to the shuttling effect of the polyiodides. Construction of artificial coatings or in situ formation of SEI films on zinc anode surfaces can inhibit the shuttle behavior of the polyiodides. Notably, in order to enhance the protective layer’s resistance to polyiodides, it can be enriched with a negative charge to inhibit the shuttling of polyiodides as well as to achieve uniform deposition of Zn^2+^. However, the current regulation of zinc anodes still has shortcomings such as complex processes and high costs. Therefore, it is necessary to explore a low-cost, industrially produced preparation process in future research to promote the development of highly stable zinc anodes.

## Figures and Tables

**Table 1:** Performance comparison of zinc–iodine batteries based on cathode material design.

Strategy Type	Cathode	Electrolyte	Separator	Specific Capacity	Cycling Stability	Refs.
Spatial confinement	ZPC/I_2_	1 M ZnSO_4_	filter paper	192 mAhg^−1^ at 0.1 A g^−1^	78.8% after 100 cycles at 0.1 A g^−1^	[54]
	BC_HP_/I_2_	1 M ZnSO_4_	GF	100 mAhg^−1^ at 0.1 A g^−1^	78.8% after 100 cycles at 0.1 A g^−1^	[55]
	CMK-3@I_2_	2 M ZnSO_4_	GF	116 mAhg^−1^ at 0.2 A g^−1^	80.6% after 39,000 cycles at 10 A g^−1^	[56]
	HCNS/I_0.5_	0.5 M ZnSO_4_ + 0.5 M H_2_SO_4_	Nafion	295.7 mAhg^−1^ at 0.5 A g^−1^	87.0% after 1500 cycles at 1 A g^−1^	[57]
	Co[Co_1/4_Fe_3/4_(CN)_6_]/I_2_	2 M ZnSO_4_	GF	236.8 mAhg^−1^ at 0.1 A g^−1^	80.2% after 2000 cycles at 4 A g^−1^	[58]
	PBI	1 M ZnSO_4_ + 0.5 mM KI	GF	242.0 mAhg^−1^ at 0.2 A g^−1^	94.0% after 1500 cycles at 4 A g^−1^	[59]
	I_2_-Nb_2_CTx MXene	1 M ZnSO_4_	GF	205.0 mAhg^−1^ at 1 A g^−1^	80.0% after 23,000 cycles at 6 A g^−1^	[40]
	Starch	Catholyte: 0.1 M I_2_ + 1 M LiI Anolyte: 0.5 M ZnSO_4_ + 0.5 M Li_2_SO_4_	GF	180.5 mAhg^−1^ at 0.2 A g^−1^	90.5% after 50,000 cycles at 10 A g^−1^	[60]
	MBene	1 M Zn(OTf) + 21 M LiTFSI	GF	201.0 mAhg^−1^ at 0.2 A g^−1^	decay ratio of 0.68% per 10,000 cycles within 23,000 cycles at 25 A g^−1^	[61]
Heteroatom Doping	I_2_/NPC-900	1 M ZnSO_4_	GF	345.5 mAhg^−1^ at 0.2 C	80.9% after 10,000 cycles at 10.0 C	[62]
	NCCs/I_2_	2 M ZnSO_4_	GF	259.0 mAhg^−1^ at 0.1 A g^−1^	100.0% after 1000 cycles at 0.1 A g^−1^	[63]
	PNC-1000-I_2_	0.05 M ZnI_2_ + 0.05 M I_2_	GF	252.0 mAhg^−1^ at 0.2 A g^−1^	89.0% after 10,000 cycles at 1.0 A g^−1^	[39]
	I_2_@NPCNFs-800	2 M ZnSO_4_	GF	228.5 mAhg^−1^ at 2.0 C	77.0% after 6000 cycles at 2.0 C	[64]
	I_2_@NHPC	1 M ZnSO_4_	GF	219.3 mAhg^−1^ at 1.0 C	decay ratio of 0.00147% per cycle within 10,000 cycles at 5.0 C	[65]
Electrostatic Interactions	GC-PAN/I	2 M ZnSO_4_	GF	146.1 mAhg^−1^ at 1.0 C	97.24% after 2000 cycles at 20 C	[47]
	PANI-I_2_	2 M ZnSO_4_	GF	230.0 mAhg^−1^ at 0.3 A g^−1^	79.0% after 700 cycles at 1.5 A g^−1^	[66]
	G/PVP@ZnI_2_	2 M ZnSO_4_ + 5 mM ZnI_2_ + 10 mM I_2_	GF	145.6 mAhg^−1^ at 0.2 A g^−1^	80.0% after 1000 cycles at 1.0 A g^−1^	[67]
Introduction of Electrocatalysts	Fe SAC-MNC/I_2_	2 M ZnSO_4_ + 0.04 M I_3_^−^	GF	188.2 mAhg^−1^ at 0.3 A g^−1^	79.5% after 50,000 cycles at 5.0 A g^−1^	[38]
	SACu@NKB	Catholyte: 0.1 M I_2_ + 1 M LiI Anolyte: 0.5 M ZnSO_4_ + 0.5 M Li2SO_4_	GF	212.2 mAhg^−1^ at 0.2 A g^−1^	92.5% after 5000 cycles at 5.0 A g^−1^	[68]
	NiSAs-HPC/I_2_	2 M ZnSO_4_	GF	202.0 mAhg^−1^ at 0.5 C	decay ratio of 0.00049% per cycle within 10,000 cycles at 20.0 C	[69]
	I_2_/Fe-NCF-700–500	2 M ZnSO_4_	GF	214.0 mAhg^−1^ at 2.0 C	79.0% after 1500 cycles at 2.0 C	[70]
	I_2_@W_2_N/N-C	2 M ZnSO_4_	GF	235.0 mAhg^−1^ at 10.0 C	85.0% after 2000 cycles at 5.0 C	[71]

**Table 2:** Performance comparison of zinc–iodine batteries based on electrolyte optimization.

Strategy Type	Cathode	Electrolyte	Separator	Specific Capacity	Specific Stability	Refs.
Electrolyte Additives	I_2_@activated carbon	2 M ZnSO_4_ + 0.2M [EMIM][OAc]	GF	223.61 mAh g^−1^ at 0.4 A g^−1^	decay ratio of 0.01% per cycle after over 18,000 cycles at 4 A g^−1^	[114]
	HOPG	ZnI_2_ + ZnSO_4_/ H_2_O + EG	filter paper	388.8 mAh cm^−3^ at 1 A g^−1^	97.6% after 15,000 cycles at 5 A g^−1^	[115]
	I_2_/ACC	1 M ZnSO_4_ with VS	GF	311 mAh g^−1^ at 0.2 C	91.0% after 13,000 cycles at 5 C	[116]
	Super P	Anolyte: Saturated pyridine−2 M ZnSO_4_ electrolyte Catholyte: 0.1 M I_2_ and 1 M LiI	GF	165.8 mAh g^−1^ at 0.2 A g^−1^	92% after 10,000 cycles at 2 A g^−1^	[45]
		Anolyte: Saturated imidazole−2 M ZnSO_4_ electrolyte Catholyte: 0.1 M I_2_ and 1 M LiI	GF	164.1 mAh g^−1^ at 0.2 A g^−1^	over 25,000 cycles at 10 A g^−1^	
Eutectic Electrolytes	AC	1 M Zn(CF_3_SO_3_)_2_/4 M N-methylacetamide/0.5 M KI in 20% volume fractions of H_2_O	glass microfiber filters	2.81 mAh cm^−2^ at 1 mA cm^−2^	98.7% after 5000 cycles at 4 mA cm^−2^	[117]
	I_2_@PC	ZnSO_4_/PG /H_2_O	-	210 mAh g^−1^ at 1 C	97.9% after 2000 cycles at 5 C	[118]
	NH_4_V_4_O_10_-PAC	EG-H_2_O-Zn[CF_3_SO_3_]_2_-ZnI_2_	glass microfiber filters	0.507 mAh at 0.2 A g^−1^	∼80% after 500 cycles at 0.2 A g^−1^	[119]
	I_2_@C	NA + DMS + Zn(ClO_4_)_2_·6H_2_O	GF	395 mAh g^−1^ at 1 A g^−1^	80% after 2000 cycles at 2 A g^−1^	[120]
Quasi-solid Gel Electrolytes	PCM−NP/I_2_	Iota-carrageenan gel electrolyte	GF	242 mAh g^−1^ at 0.5 C	91.9% after 5000 cycles at 5 C	[121]
	I_2_@AC	Alginate-Based Hydrogel	GF	196.4 mAh g^−1^ at 0.1 A g^−1^	66.8% after 2000 cycles at 2 A g^−1^	[122]
	Carbon cloth	Anolyte: 0.5 M ZnSO_4_/PEO_53_–PPO_34_–PEO_53_; catholyte: 0.1 M I_2_/1 M KI/PEO_53_–PPO_34_–PEO_53_	-	252 mAh g^−1^ at 0.1 C	94.3% after 500 cycles at 1 C	[123]

**Table 3:** Performance comparison of zinc–iodine batteries based on diaphragm modification.

Cathode	Electrolyte	Separator	Specific Capacity	Specific Stability	Refs.
MPC/I_2_	1 M ZnSO_4_	KB@CF	142 mAh g^−1^ at 0.1 A g^−1^	average capacity decay rate of 0.030% per cycle over 2000 cycles at 1 A g^−1^	[137]
KB	Catholyte: 0.5 M ZnSO_4_, 1 M LiI, and 0.1 M I_2_ Anolyte: 0.5 M ZnSO_4_ and 0.5 M Li_2_SO_4_	Zn–BTC	203 mAh g^−1^ at 160 mA g^−1^	84.6% after 6000 cycles at 1.92 A g^−1^	[138]
I_2_/ACC	1 M ZnSO_4_	Dowex + Fe–SCNT/GF	1.72 mAh cm^−2^ at 0.2 A g^−1^	average capacity decay of 0.0008% per cycle at 5 A g^−1^ after more than 30,000 cycles	[139]
KB	Anolyte: 0.5 M ZnSO_4_/0.5 M Li_2_SO_4_; catholyte: 0.5 M ZnSO_4_/1 M LiI/0.1 M I_2_	Zeolite membrane of Na_12_(AlO_2_)_12_(SiO_2_)_12_·xH_2_O	123.4 mAh g^−1^ at 0.2 A g^−1^	91.0% after 30,000 cycles at 4 A g^−1^	[140]

**Table 4:** Performance comparison of zinc–iodine batteries based on zinc anode modification.

Cathode	Anode	Electrolyte	Separator	Specific Capacity	Specific Stability	Refs.
GC-PAN/I	SC-PPS@Zn	2 M ZnSO_4_	GF	130.4 mAh g^−1^ at 1 C	90.2% after over 6000 cycles at 3.2 A g^−1^	[33]
KB	Zn@Ts	Anolyte, 0.5 M Li_2_SO_4_/0.5 M ZnSO_4_; catholyte, 1 M LiI/0.1 M I_2_/0.5 M ZnSO_4_	GF	150 mAh g^−1^ at 0.4 A g^−1^	93% after 20,000 cycles at 6 A g^−1^	[146]
I_2_/N, P co-doped carbon	PA−Zn	2 M ZnSO_4_	GF	261.1 mAh g^−1^ at 0.5 C	5000 cycles at 5 C	[147]
I_2_@AC	Zeolite−Zn discs	1 M ZnSO_4_	GF	203−196 mAh g^−1^ at 0.2 A g^−1^	91.92% after 5600 cycles at 2 A g^−1^	[148]

## Data Availability

The authors do not have permission to share data.
